# Evaluating the Efficiency of Black Soldier Fly (*Hermetia illucens*) Larvae in Converting Mackerel Head Waste into Valuable Resources

**DOI:** 10.3390/ani14091332

**Published:** 2024-04-29

**Authors:** Gabriel Tirtawijaya, Jin-Hwa Lee, Khawaja Muhammad Imran Bashir, Hae-Jeung Lee, Jae-Suk Choi

**Affiliations:** 1 Faculty of Biotechnology, University of Surabaya, Jalan Raya Kalirungkut, Surabaya 60292, Indonesia; gabrieltirtawijaya@staff.ubaya.ac.id; 2 Department of Seafood Science and Technology, The Institute of Marine Industry, Gyeongsang National University, Tongyeong 53064, Republic of Korea; evolution_5237@gnu.ac.kr (J.-H.L.); imranbashir@gnu.ac.kr (K.M.I.B.); 3 German Engineering Research and Development Center for Life Science Technologies in Medicine and Environment, Busan 46742, Republic of Korea; 4 Department of Food and Nutrition, College of BioNano Technology, Gachon University, Seongnam 13120, Republic of Korea

**Keywords:** alternative feed, black soldier fly larvae, biomass conversion, insect farming, waste management, waste-to-biomass

## Abstract

**Simple Summary:**

This study addresses the challenges of waste generated in ready-to-eat (RTE) food production, with a focus on mackerel processing. By-products such as head and intestine are often discarded as waste, prompting the investigation of an eco-friendly solution using black soldier fly larvae (BSF larvae) as bio-converters. In this study, BSF larvae efficiently transformed mackerel heads (MH) into valuable biomass, with the most substantial growth occurring when BSF larvae were fed a diet containing 20% MH waste. Beyond this concentration, the growth rate declined, indicating an optimal waste conversion point. These findings hold societal value on multiple fronts. Firstly, this study introduces an innovative and eco-friendly approach for seafood industry waste management by converting MH waste into valuable biomass and reducing the environmental impact of waste disposal. Furthermore, the research underscores the potential of BSF larvae as a sustainable alternative animal feed source, potentially decreasing reliance on conventional feeds. In conclusion, this study offers a practical solution for waste management and sustainable food production, aligning with broader efforts to establish an eco-friendly and efficient food industry.

**Abstract:**

The seafood processing industry generates significant waste, including mackerel heads (MH), constituting 20–32% of total waste. This study explored the potential of utilizing MH as a feed source for black soldier fly larvae (BSF larvae). BSF larvae are known for their ability to efficiently convert organic materials into nutrient-rich biomass. Five concentrations of MH (0, 10, 20, 30, 40, and 50% in chicken feed) were fed to BSF larvae for eight days. After harvesting, their growth, MH conversion efficiency, nutritional content, and heavy metals reduction potential were measured. BSF larvae showed optimal growth when fed with a feed containing 20% MH, resulting in a 14.36-fold increase in weight compared to the control group, as determined by the Fisher’s Least Significant Difference Test. BSF larvae maintained a survival rate of 99.33%. With the lowest feed conversion ratio (FCR) of 2.09 at 20% MH, feed efficiency was improved by up to 65.15%, and feed reduction up to 73.53%. MH enhanced lipid and protein content in BSF larvae. Furthermore, BSF larvae in this study showed higher polyunsaturated fatty acids (PUFA), including eicosapentaenoic acid (EPA) and docosahexaenoic acid (DHA), as well as other amino acids which are required for breeding animals. The current study highlights the potential of MH as a feed source for BSF larvae, improving nutritional biomass. It also suggests BSF larvae as an eco-friendly option for handling seafood processing waste and as an alternative feed source for animals.

## 1. Introduction

The development of the ready-to-eat (RTE) food processing industry has resulted in an increase in waste production. Mackerel is a fishery commodity that is increasingly used in RTE food processing. Mackerel is industrially prepared by various processes, such as roasting, smoking, salting, and canning [1,2]. The by-products of mackerel processing comprise intestine and head, and these potentially end up as waste unless they are utilized. Fish head waste produced from fish processing accounts for 20–32%, and it is usually considered an inedible part [3,4]. Mackerel is one of the favorite fish species in the Republic of Korea; however, unlike some other countries where fish by-products are utilized in bait or fishmeal [5], its head is typically not consumed in Korea and often ends up as fish waste [6]. Ramakrishnan et al. [7] reported that mackerel head (MH) comprises 36.8% of total fish processing waste. Since a significant amount of fish waste is produced, it is necessary to develop an alternative management strategy to reuse this material. The utilization of fish head waste provides a new opportunity as a feed source for black soldier fly larvae (BSF larvae).

BSF larvae are known to recycle organic material into biomass with good nutritional content. The use of BSF larvae provides an environmentally friendly and inexpensive alternative to waste treatment [8]. Recently, rearing of BSF larvae on organic materials has been extensively reported; for example, feeding on household waste [9,10], agri-food leftovers [11,12], animal farm waste [13,14,15], and fishery waste [16,17]. Different organic materials employed as substrates in BSF larvae cultivation generate distinct nutritional content; for instance, a high-protein substrate produces high-protein BSF larvae [18]. In the wild, BSF larvae do not contain polyunsaturated fatty acids (PUFA), such as eicosapentaenoic acid (EPA) and docosahexaenoic acid (DHA). However, they may contain PUFA after consuming foods rich in PUFA [19].

The accumulation of heavy metals in BSF larvae and its impact on their growth and development has been extensively documented [20,21,22,23,24]. Organic waste often contains persistent contaminants, including heavy metals, which can accumulate in BSF larvae and pupae, potentially infiltrating the broader food chain [20]. While terrestrial organisms ingest contaminants orally (biomagnification), aquatic organisms accumulate pollutants through diffusion (bioconcentration) [20]. Bioaccumulation refers to both biomagnification and bioconcentration [25]. The bioaccumulation factor, therefore, represents the concentration of a pollutant in organisms divided by its concentration in the diet [20]. Numerous fish species, including mackerel, tuna, and bonitos, have been reported to accumulate heavy metals in their tissues [26,27]. For instance, in a study examining the mercury levels in king mackerel, concentrations ranged from 0.19 to 3.6 ppm in mackerel collected from the Atlantic coast and from 0.18 to 4.0 ppm in those from the Gulf coast. Similarly, total mercury levels in Spanish mackerel ranged from 0.04 to 1.3 ppm from the Atlantic coast and from 0.09 to 3.2 ppm from the Gulf coast [26]. Therefore, in this study, we also investigated heavy metal accumulation in BSF larvae.

BSF larvae are rich in protein and lipid content, containing up to 40.87–49.18% and 26.67–38.67%, respectively [9,11,14]. Leveraging their nutritional richness, numerous studies have explored the application of BSF larvae as an alternative to conventional feeds in both livestock and aquaculture [19,28,29,30]. Consequently, BSF larvae emerge as a promising bioconversion agent for organic waste, presenting an alternative feed source. However, existing research on utilizing fishery waste as a substrate for BSF larvae has primarily focused on aspects such as fatty acid alteration and growth performance [16,17,31]. In a study by St-Hilaire et al. [16], an increase in omega-3 fatty acid content was observed by feeding fish offal and cow manure to BSF larvae; however, specific information regarding fish species and parts used was not provided. Similarly, Barroso et al. [17] explored the use of discarded round sardinella (*Sardinella aurita*) as fish waste feed for BSF larvae, investigating the impact of consuming an omega-3 fatty acid-rich fish waste on the fatty acid profile of BSF larvae. Nevertheless, neither study specified the fish species’ parts, and the fatty acid composition of fish offal was not examined. It is noteworthy that fish exhibit varying fatty acid content and composition depending on the species and part. In contrast to prior studies utilizing fish waste [16,17], we focused exclusively on feeding the head part of a specific fish species, mackerel, to BSF larvae—a novel approach, to the best of our knowledge, not reported before. Additionally, we investigated the fatty acid content and heavy metal accumulation in BSF larvae fed on the prepared diet. Furthermore, this study assessed the waste conversion efficiency and waste reduction potential of BSF larvae for the management of fishery waste, particularly MH waste. This study introduces an innovative strategy for efficiently converting MH waste into valuable biomass using BSF larvae.

## 2. Materials and Methods

### 2.1. Preparation and Selection of Animals

Two-day hatched black soldier fly, *Hermetia illucens* (L.) (Diptera: Stratiomyidae), larvae were obtained from AgroK Corporation, Busan, Republic of Korea. Chicken feed (CF; Hanyang Feed Co., Ltd., Seoul, Republic of Korea) was offered ad libitum to BSF larvae for two days before selection of experimental subjects. BSF larvae with an average weight of 6.5 ± 1 mg were selected using mesh no. 10 (2 mm pore size) and one day fasted before experiment. The larvae were also subjected to fasting at the end of the experiment to avoid the influence of substrate content in the larval digestive system on further analyses. Through this fasting, measurements of the initial and final weights of the experimental larvae were conducted under similar conditions.

### 2.2. Preparation of Mackerel Head and Larvae Feed

The mackerel heads (MH) were procured from the local Mackerel Foods Corporations, situated in Busan, Republic of Korea (GPS coordinates: 35°03′20″ N 129°00′39″ E). Initially, the MH was stored at −20 °C before utilization in the experiment. To prepare the MH for experimentation, the frozen MH were first chopped and then pulverized into an icy slurry form. This icy slurry of MH underwent filtration through mesh no. 18 (1 mm aperture size) to remove undesired materials, such as bones and uncrushed parts. The control diet (CF) was prepared by pulverizing and sieving CF powder through a 425 µm aperture size. CF (100%) was used as a control diet, while other experimental treatments involved a mixture of CF with varying proportions of MH (10, 20, 30, 40, and 50%). Water was added to the feed mixture to achieve a final moisture content of approximately 60%. 300 g of feed was prepared for each treatment group. The feed mixtures were stored in sealed containers at −20 °C until further use. Before feeding the BSF larvae, the feed mixtures were thawed to room temperature and homogenized. For the proximate composition and fatty acid content of the feed used in this study, refer to Table 1 and Table 2, respectively.

### 2.3. Rearing Conditions and Growth of BSF Larvae

BSF larvae rearing conditions for measuring survival rate (SR), growth, and feed conversion efficiency were established based on our previous study [32]. Two separate experiments were conducted to evaluate the impact of MH on these parameters. The first experiment focused on SR, using 200 larvae per 20 cm^2^ box, and lasted for eight days. The second experiment evaluated growth and feed conversion efficiency, utilizing 600 larvae in 115 cm^2^ jars, also for eight days. In both experiments, the larvae were provided with an equivalent amount of feed relative to their weight. The rearing containers, consisting of a total of 18 boxes and jars, were covered with nylon nets and kept at a temperature of 27 ± 1 °C and a relative humidity ranging from 40% to 50%. These containers were divided into six treatment groups, with each group containing three replications (*n* = 3). After 8 days of culturing, BSF larvae reached 4th instar.

During the feeding period of seven days, BSF larvae were given MH at varying concentrations: 0, 10, 20, 30, 40, and 50%. The daily feed amounts increased progressively throughout the experiment, commencing at 10 g per day on days 1 to 3, escalating to 20 g per day on days 4 and 5, and culminating at 25 g per day on days 6 and 7. The feed rations were incrementally adjusted based on initial observations to maintain optimal sustenance levels for the larvae, avoiding both overfeeding and underfeeding. This feeding regimen resulted in a total of 120 g of feed per treatment for evaluating growth and feed conversion efficiency. On day eight, the BSF larvae were removed from the rearing jars, and any substance adhering to their bodies was separated and collected. This separated substrate was oven-dried at 50 °C until constant weight was achieved and was used to determine the remaining feed (residue). After measuring the water content of the feed at the experiment’s outset, the dry weight of the feed residue was converted to wet weight for calculating the efficiency of ingested feed (ECI) and feed reduction (FR). For the evaluation of ECI, the difference in wet weight of larvae on day 0 and day 8 was divided by the wet weight of the remaining feed. The BSF larvae were then rinsed with tap water, dried with paper towels, and weighed to assess their growth increase (GI) and feed conversion ratio (FCR). For the calculation of the FCR, the wet weight of the total provided feed was divided by the difference in wet weight of larvae on day 0 and day 8. Subsequently, the BSF larvae were frozen at −18 °C for 3 h before being oven-dried at 50 °C for three days. The dried BSF larvae were pulverized and stored at −18 °C until further analysis. In these experiments, the following equations (Equations (1)–(5)) were employed for calculations.

(1) $$\mathrm{GI}\;(x\;\mathrm{fold})=\frac{W_{8}-{\;W}_{0}}{W_{0}}$$

(2) $$\mathrm{SR}\;(\%)=\left(\frac{N_{8}}{N_{0}}\right)\times 100$$

(3) $$\mathrm{FCR}=\left(\frac{F}{W_{8}-{\;W}_{0}}\right)$$

(4) $$\mathrm{ECI}\;(\%)=\left(\frac{W_{8}-{\;W}_{0}}{F-R}\right)\times 100$$

(5) $$\mathrm{FR}\;(\%)=\left(\frac{F-R}{F}\right)\times 100$$

where W_8_ represents the wet weight of all BSF larvae per group treatment on day 8, W_0_ signifies the wet weight of all BSF larvae per group treatment on day 0, N_8_ denotes the total number of BSF larvae on day 8, N_0_ represents the total number of BSF larvae on day 0, F stands for the total amount of provided feed, and R signifies the total amount of remaining feed.

### 2.4. Nutritional Content Analysis

The nutritional content of BSF larvae was calculated by measuring protein, lipid, ash, amino acids, and fatty acid content in BSF larvae. The protein, lipid, and ash content were measured following the Official Methods of Analysis (OMA) of the Association of Official Analytical Collaboration (AOAC) International [33]. The amino acid composition was measured using an amino acid analyzer (Hitachi L-8900; Hitachi High-Tech Corp., Tokyo, Japan). The fatty acid content was measured using a GCMS Shimadzu GC-2010 Plus with FID (Shimadzu Corp., Kyoto, Japan) after methylation of crude lipids [32].

### 2.5. Heavy Metal Bioaccumulation Analysis

The degree of metal accumulation in BSF larvae fed with chicken feed containing 0 to 50% (*w*/*w*) mackerel by-products was analyzed. Heavy metals such as arsenic (As), cadmium (Cd), and lead (Pb) were measured according to the method mentioned in the Food Code [34] using inductively coupled plasma optical emission spectroscopy—ICP OES Optima 8300 (PerkinElmer Inc., Billerica, MA, USA). The total mercury (Hg) content was analyzed using the EPA 7473 method [35] with a Hydra IIc Mercury Analyzer (Teledyne Leeman Labs, Mason, OH, USA). In addition, the heavy metal reduction percentage (MR%) in BSF larvae was calculated using Equation (6).

(6) $$\mathrm{MR}\;\left(\%\right)=\left(\frac{\left(M_{\mathrm{BSFL}}-M_{\mathrm{SBSFL}}\right)}{\left(M_{\mathrm{BSFL}}-M_{\mathrm{SBSFL}}\right)+\left(M_{\mathrm{TF}}-M_{\mathrm{BSFLD}}\right)}\right)\times 100$$

where M_BSFL_ means the heavy metals in BSF larvae that consumed chicken feed containing 0 to 50% (*w*/*w*) mackerel head, fasted, and with feces removed; M_SBSFL_ indicates heavy metals in BSF larvae before starting the feeding experiments; M_TF_ indicates heavy metals in chicken feed supplemented with 0 to 50% (*w*/*w*) mackerel head; M_BSFLD_ means heavy metals in the feces of BSF larvae.

### 2.6. Statistical Analysis

Data are shown as means ± standard error (S.E.) of three replicates in each group (*n* = 3). Statistical analyses were performed using one-way analysis of variance (ANOVA) and statistical significance was estimated using IBM SPSS Statistics ver. 23 (IBM Corp., New York, NY, USA). The post hoc tests were performed using the Fisher’s Least Significant Difference (LSD) test and the values were considered statistically significant at *p* < 0.05.

## 3. Results

### 3.1. Growth Performance of MH-Treated BSF Larvae

**Figure 1:**
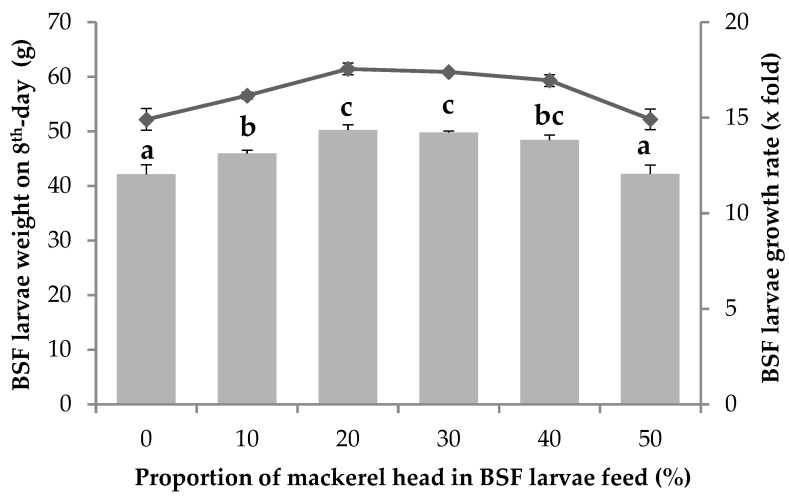
Growth performance of black soldier fly larvae (BSF larvae) treated with mackerel head. Values are expressed as means ± S.E. of three replicates (*n* = 3). A line graph illustrates changes in BSF larvae weight on the 8th day, and the growth rate is represented in a column graph. Different lowercase letters (a–c) indicate significant differences in growth rate among treatments at *p* < 0.05, according to the LSD test.

The growth performance data of BSF larvae fed with feed supplemented with MH is illustrated in Figure 1. The results indicate a noteworthy trend: as the concentration of MH increased, the growth of BSF larvae exhibited a corresponding increase, up to a concentration of 20%. These findings were accompanied by a significant difference among the treatment groups (*p* < 0.05), implying a substantial impact of MH addition to the diet on the growth performance of BSF larvae. Post hoc analysis unveiled specific patterns of significance. In particular, the most striking increase in growth occurred in the MH 20% treatment group, which demonstrated a remarkable 14.36-fold increase in weight, transitioning from 4 g to 61.45 g on the 8th day of rearing. Importantly, this surge in growth was statistically significant when compared to the control group (MH 0%). The treatment groups receiving 30% and 40% MH also displayed significant growth increases compared to the control group (*p* < 0.05). However, their growth, while notable, was still less than that of the 20% MH group, although this difference was not statistically significant (*p* < 0.05). Furthermore, the BSF larvae growth remained similar to the control group receiving the basal feed when provided with 50% MH (*p* < 0.05). This observation suggests that MH proportions in the BSF larvae feed exceeding 20% do not effectively promote BSF larvae growth.

### 3.2. The Efficacy of BSF Larvae in Converting MH

The BSF larvae’s survival rate, FCR, ECI, and FR were calculated to evaluate their response to variations in MH concentrations (Table 3). The results indicate that BSF larvae exhibit a high survival rate when provided with MH-containing feed. The survival rates were consistently above 80%, indicating the resilience and adaptability of BSF larvae in converting the organic waste. The survival rates in the control group (0% MH) and MH (up to 20%) showed no significant differences (*p* > 0.05). This shows that BSF larvae can efficiently consume MH up to a level of 20%, as evidenced by a reduction in growth at this level (Figure 1).

FCR represents the ratio of feed consumed by BSF larvae to the weight gained during the conversion process. Notably, the addition of MH at concentrations ranging from 10% to 40% had a significant impact on the FCR value (*p* < 0.05) compared to 0% or 50% MH. At an MH concentration of 0%, the FCR value was 2.50, indicating that BSF larvae required 2.50 g of feed to gain 1 g of biomass. As the proportion of MH in the BSF larvae’s feed increased, the FCR demonstrated a consistent trend of improvement. Specifically, at an MH concentration of 10%, the FCR decreased to 2.28, signifying a more efficient conversion of feed into biomass. This trend continued, and at MH 20%, the FCR further reduced to 2.09, indicating higher feed efficiency. Interestingly, at MH 30%, the FCR remained relatively stable at 2.11. However, as the MH proportion increased to 40% and 50%, the FCR slightly increased again to 2.17 and 2.50, respectively. Despite the lack of statistically significant differences in FCR between MH concentrations of 10% to 40% (*p* < 0.05), the BSF larvae exhibited more efficient utilization of MH at proportions of 10% to 30% compared to 0% or 50% MH.

The ECI measurements provide a deeper understanding of BSF larvae feed conversion efficiency for optimal growth. At MH 0%, the ECI of the BSF larvae was 57.56%. This means that the BSF larvae efficiently converted 57.56% of ingested feed into biomass. ECI values significantly increased with increasing proportion of MH in BSF larvae feed (*p* < 0.05). BSF larvae efficiently converted MH into biomass up to 40% MH, and after that the ECI value began to decrease. BSF larvae with the lowest amount of unconsumed feed showed the highest waste reducing efficiency, as indicated by a high FR value. At MH 0%, the FR of the BSF larvae was 69.75%, indicating that the BSF larvae did not consume approximately 30.25% of their feed. The FR value increased significantly with increasing proportion of MH up to a level of 30% MH (*p* < 0.05). At MH 40 and 50%, FR decreased significantly to even less than the control group, to 66.94% and 65.08%, respectively. This indicates that at higher proportions of MH in feed, the BSF larvae were no longer efficient in reducing feed.

### 3.3. Proximate Composition of MH-Treated BSF Larvae

Considering the diverse applications of BSF larvae and their biomass, we conducted an evaluation of the proximate composition of MH-treated BSF larvae. This evaluation included an analysis of lipid, protein, and ash content, which is summarized in Table 4. The presence of MH in basal feed significantly increased lipid and protein content; however, it decreased the ash content (*p* < 0.05). The highest lipid content was obtained with feed containing 40% MH as substrate, which insignificantly differed from MH 20%. A similar trend was observed in protein content, which was highest in 50% MH but differed insignificantly to the treatment group fed with feed containing 20% MH. The control group substrate had the greatest ash level, which decreased dramatically in a concentration-dependent manner.

The nutritional content of MH-treated BSF larvae was thoroughly evaluated in terms of fatty acids and amino acid content. Table 5 shows the fatty acid composition of BSF larvae fed with different concentrations of MH substrate. The lauric acid, heneicosanoic acid, and linoleic acid content decreased in MH-fed BSF larvae compared to the BSF larvae fed with basal feed (CF). The myristic acid, pentadecanoic acid, palmitic acid, margaric acid, palmitoleic acid, eicosaenoic acid, and linolenic acid content increased with increasing proportion of MH in BSF larvae feed. MH presence in basal feed also contributed to tridecanoic acid, linoelaidic acid, γ-Linolenic acid, arachidonic acid, EPA, and DHA content in BSF larvae, and showed a rising trend with increasing MH concentration. The saturated fatty acid content (55.58–57.15%) in MH-fed BSF larvae was lower than the control (60.09%), whereas the unsaturated fatty acid (monounsaturated fatty acid—MUFA and PUFA) content increased in MH-treated BSF larvae.

The amino acid composition in g/kg dry matter (D.M.) of MH-treated BSF larvae is listed in Table 6. High concentrations of non-essential amino acids, such as glutamic acid (45.6–47.2 g/kg D.M.) and alanine (40.5–43.7 g/kg D.M.) was observed in BSF larvae. Concurrently, the following most abundant composition consisted of essential amino acids, specifically leucine (26.6–27.4 g/kg D.M.), lysine (23.8–25.8 g/kg D.M.), and valine (23.6–24.4 g/kg D.M.). The differences in amino acid composition of BSF larvae fed on 0, 20, and 50% of MH were negligible. Apparently, the variation in MH proportion in BSF larvae feed did not result in a visible effect on amino acid content variation in BSF larvae.

### 3.4. Heavy Metal Reduction Potential of BSF Larvae

The analysis of heavy metals in the standard CF used in this study revealed concentrations of 0.143 ± 0.001, 0.065 ± 0.011, 0.010 ± 0.001, and 0.005 ± 0.001 mg/kg for Pb, As, Hg, and Cd, respectively. In comparison, MH samples showed concentrations of 0.046 ± 0.001, 0.203 ± 0.081, 0.030 ± 0.001, and 0.041 ± 0.001 mg/kg for Pb, As, Hg, and Cd, respectively (Table 7). BSF larvae, after removing intestinal feces, exhibited Pb, As, Hg, and Cd contents of 0.263 ± 0.009, 0.253 ± 0.015, 0.0054 ± 0.008, and 0.758 ± 0.062 mg/kg, respectively.

The heavy metal content in BSF larvae which consumed CF containing 0 to 50% (*w*/*w*) of mackerel by-products, fasted, and had feces removed, ranged from 0.048 ± 0.007 to 0.148 ± 0.006 mg/kg for Pb, 0.025 ± 0.043 to 0.352 ± 0.143 mg/kg for As, 0.004 ± 0.002 to 0.049 ± 0.007 mg/kg for Hg, and 0.086 ± 0.032 to 0.132 ± 0.016 mg/kg for Cd (Table 7). A notable increase in As and Hg content was observed in BSF larvae fed with feed containing 50% MH compared to 0%, indicating a rising trend in heavy metal content in BSF larvae corresponding to the feeding ratio of MH. These results were confirmed to be within the standards ratios and specifications for ‘fisheries products’ mentioned in the April 2017 re-evaluation report on heavy metal standards for food [28].

The BSF larvae exhibited a metal reduction percentage ranging from 44 to 65% for Pb and 366 to 430% for Cd when fed with 0–50% MH (Table 7). The reduction percentage for As ranged from 38 to 97% when BSF larvae were fed with 0–30% MH; however, BSF larvae failed to reduce As content when fed with feed containing a higher proportion of MH (>30%). In contrast, only a minor metal reduction was observed for Hg, ranging from 3 to 20%.

## 4. Discussion

In recent years, insects have gained prominence as a promising source of sustenance [37,38]. Utilizing insects for feed and food production is increasingly acknowledged as a viable strategy to meet the food demands of a growing population. Unlike conventional animal production, farming insects as mini-livestock leads to lower emissions of ammonia and greenhouse gases [39]. Insect meals are being recognized as an important alternative due to their high nutritional value [40]. Furthermore, insects demonstrate high feed conversion efficiency, likely due to their poikilothermic physiology [37]. Among insects, the utilization of BSF larvae is gaining recognition for their potential in agriculture and waste management, as they effectively convert organic waste into protein-rich biomass [41]. Additionally, BSF larvae are rich in calcium (5–8% dry matter), a crucial element for fish species such as crayfish, aiding in the formation of their new exoskeleton after molting [42,43]. Extensive research has focused on exploring the suitability of BSF larvae as a source of nourishment for various animals including fish, poultry, and pigs [43,44,45]. In this study, we investigated the nutritional profile and feed conversion efficiency of BSF larvae fed on fisheries waste, especially ‘mackerel head—MH’.

The current study explored BSF larvae growth performance under different proportions of MH in their feed and observed the resulting patterns. The addition of MH to BSF larvae substrate resulted in a higher increase in growth compared to BSF larvae fed on basal feed (CF), particularly at MH concentrations of 20–40%. Previous studies have also described the use of fishery waste for developing BSF larvae [16,17,31,46]. Pérez-Pacheco et al. [46] reported the development and growth characteristics of BSF larvae fed with *Solea solea* L. fish, showing a shortened development time, though this was not better than feeding on restaurant waste in terms of growth. The restaurant waste used in their study comprised up to 80–85% carbohydrate from corn tortillas, rice, and cooked tomatoes, and protein and fat from chicken meat, pork, and beef. This is likely to be linked to an imbalance in the feed content required for BSF larvae growth. Therefore, in the present study, the effect of fish processing waste (MH) was investigated to optimize BSF larvae growth. Arena et al. [31] reported significantly higher growth performance of BSF larvae fed on control feed (75% peat and 25% wheat bran) than on various fishery wastes (25%), which could be due to different availability of carbon and degradable nutrients in the feeding substrate.

Higher MH proportion in BSF larvae feed increases protein and fat content while decreasing carbon sources. Since digestible carbohydrate content is the main source of energy for most insect species including BSF larvae [47], it is essential for optimal growth [48]. The BSF larvae reared on high carbohydrate-content feed (55%) showed higher yields than low carbohydrate content (35–45%), although they contained varying amounts of protein (10–24%) [49]. This suggests that protein content in feed is required for BSF larvae development, as it contains a sufficient amount of carbohydrates. Several studies have demonstrated that the development of BSF larvae is affected by the ratio of protein to carbohydrates in their diet. A high protein content in the feed leads to increased protein content in the BSF larvae and faster development, but it results in lower larval mass [50]. Eggink et al. [51] found that the optimal protein to carbohydrate ratio for the highest larval yield is between 1:2 and 1:3. The present study utilized the by-difference approach to determine the carbohydrate content of the feed. The highest mass production of BSF larvae was observed in the MH 20% treatment, with a protein-to-carbohydrate ratio of 1:1.8. These findings suggest that the specific treatment conditions and nutrient composition of the feed may have a significant impact on the growth performance of BSF larvae. Nguyen et al. [52] reported that BSF larvae, when fed with 100% fish waste, experienced elevated mortality rates of up to 52.77%, along with reduced weight and length compared to the control group (CF). This outcome may be attributed to the excessive fat content in fish waste, which proved detrimental to the well-being of BSF larvae [52]. As a result, we constrained the mixing ratio to a range of 10% to 50% for MH and CF. This adjustment aimed to ensure the substantial accumulation of omega-3 fatty acids without causing a significant decline in the survival rate of BSF larvae. Fish waste may also contain heavy metals that affect the survival rate of BSF larvae. Cambron et al. [53] reported that as the insect larvae achieve a needed lipid limit, they lower their feeding consumption. In the present study, increasing protein sources consequently decreased carbohydrate levels, which could have forced BSF larvae to use protein as their main source of energy. Meanwhile, the use of protein as a source of energy is less efficient and more costly than carbohydrate [54]. This explains the poor growth performance in higher MH treatment groups, particularly at 50% concentration. The highest increase in growth observed in the 20% MH treatment group indicates the appropriate amount of required protein. Although a protein-rich diet could increase growth rate, the high weight and yield of BSF larvae have been linked to superior nutritional quality and substrate heterogeneity [55].

The efficacy of BSF larvae in converting waste materials can be predicted from the FCR, ECI, FR, and SR, which are essential factors in determining BSF larvae success in waste conversion [32,55,56]. Lower FCR and higher ECI values indicate higher efficiency of BSF larvae in converting the provided feed into biomass, making them an efficient option for waste conversion. The inclusion of MH reduces the FCR, thus it is important for the production of BSF larvae as a source of feed for breeding animals. Furthermore, a high reduction efficiency of waste or feed is represented by higher FR [57]. The FCR values observed at 10–50% MH in this study ranged from 2.09–2.50, which are lower than those reported in previous studies using various feed sources such as restaurant food waste (FCR 2.6), brewer’s waste (FCR 2.7), fecal sludge (FCR 3.4), banana peelings (FCR 4.5) [55], soybean curd residue and kitchen waste (FCR 2.51) [9], fruit puree (FCR 3.19), and household food waste (FCR 6.32) [56]. The lower FCR values observed in this study indicate a more efficient feed conversion by the BSF larvae. Laganaro et al. [58] observed that substrate quality has an impact on BSF larvae growth. In the present study, the growth performance of BSF larvae fed with 20% MH was the highest, providing an optimal substrate value for significant increase in growth and a high survival rate of 99.33%.

ECI value provides an overall evaluation regarding an insect’s capacity to utilize ingested food for growth and development [10]. In addition, it also contributes to a reduced amount of feed (FR). In a recent study, we reported that BSF larvae efficiently convert ingested feed containing squid liver oil to biomass [32]. We observed an increase in growth indicated by high ECI and high FR values. Results of the present study exhibited higher ECI values obtained from feed containing 40% MH; however, the differences among concentration groups were insignificant. In addition, the highest FR value was obtained from feed containing 20–30% MH. These findings show that the BSF larvae fed with 20% MH efficiently utilized substrate (FR), converting it into biomass (ECI). FR values demonstrate the amount of waste consumed from the total waste provided. This is important to assess, since it allows for controlling the amount of waste provided to the BSF larvae. Previous studies show the percentage of waste reduction (FR) of various wastes by BSF larvae, including animal manure (12.7–60.0%) [41,59], food waste (52.3–66.7%) [41,60,61], fruits and vegetables (46.7%) [41], and municipal organic waste (68%) [62]. The obtained FR values in the present study were high, reaching 69.75% in the control group and up to 73.53% in the 20% MH treatment group. In comparison to other concentrations, the FCR and ECI values were more efficient at 20% MH. Feed quality not only affects the BSF larvae size, but also survival rate [63]. The findings of the present study indicate that feed containing 20% MH provides good quality BSF larvae, with a higher increase in growth and survival rate. However, at MH 50%, the ECI decreased to 61.88%, suggesting that the higher concentrations of MH might have a negative impact on the feed conversion efficiency, possibly due to suboptimal nutritional balance or other limiting factors. Overall, BSF larvae efficiently converted MH into valuable biomass, with MH concentrations around 20–40% being particularly efficient.

Previous research employing different feed sources revealed varied protein and lipid content in BSF larvae. The protein and lipid content reported by previous studies, however, were within a narrow range of 41.2–43.1% and 33.6–38.6%, respectively [64,65]. Increased proportion of MH in the BSF larvae feed significantly enhanced protein (41.37–42.69%) and lipid (35.80–37.74%) content while decreasing ash (6.10–4.90%). However, 20% MH resulted in a substantial increase in lipid, but not protein content, while an MH concentration of 50% resulted in the lowest ash content. Purkayastha and Sarkar [66] observed that the quality of feed or waste does not affect the level of protein content in BSF larvae; however, it affects the level of fat content. The ash content in CF has been reported at 2.07–6.30% [32,52]; meanwhile, the ash content of mackerel is 1.36–1.63% [67,68]. Given that MH has relatively lower ash content than CF, increasing the concentration of MH may cause a decrease in the feed substrate’s ash level. Overall, the ash content of BSF larvae in the present investigation showed a variety of values, as previously reported (4.98–7.91%) [17,32].

One interesting characteristic of the MH-containing feed is the presence of increased PUFA content as well as the existence of arachidonic acid, EPA, and DHA in BSF larvae. EPA and DHA are usually found in BSF larvae reared on marine-based substrate, such as fishery waste, macroalgae, and oil from fish or squid [16,17,32,69,70]. Barroso et al. [17] reported a 3-fold increase in EPA and DHA in BSF larvae after one day ad libitum feeding with *Sardinella aurita*. CF supplemented with 5% squid liver oil also increased PUFA content by 16.27%, EPA by 2.99%, and DHA by 2.68% in BSF larvae [18]. BSF larvae treated with 20% MH showed higher PUFA (17.79%) and EPA (3.19%), and lower DHA (1.74%) content. BSF larvae reared on CF, mixed feed, and brewer’s spent grain did not show the presence of EPA and DHA because there was no EPA and DHA in these substrates [71]. BSF larvae fed with substrate containing EPA and DHA, such as mussels, rapeseed cake, and shrimp waste has shown higher EPA and DHA content [71]. The amount of PUFA present in feeding substrate determines the level of EPA and DHA in BSF larvae [17]. Therefore, the use of MH as a substrate for BSF larvae could enhance their nutritional value and suitability as a feed source by increasing the accumulation of PUFA.

The amino acid composition of BSF larvae fed with MH at 20% and 50%, as well as the control group, did not exhibit any particular trend. These findings align with previous studies by Wang et al. [65] and Oonincx and Finke [72], suggesting that amino acid content in BSF larvae is not significantly affected by their dietary intake. However, it is important to note that the amino acid composition observed in this study contradicts the previous studies [65,72], particularly in the case of amino acids such as alanine, arginine, aspartic acid, cysteine, serine, threonine, tryptophan, and valine. Wang et al. [65] suggested that these differences might be attributed to variations in the geographical strain of BSF larvae and measuring techniques. The amino acid content may also differ due to variations in the analytical methods employed. The tryptophan content of BSF larvae in this study (3.5–4.2%) was only half of that reported in the study by Eggink et al. [71] (7.1–8.0%). Eggink et al. [71] conducted a comprehensive analysis of all amino acids, excluding tryptophan, using High-Performance Liquid Chromatography (HPLC), while specifically analyzing tryptophan using a spectrophotometer. Separate analyses were performed to mitigate concerns regarding tryptophan degradation during acid hydrolysis in the sample preparation. The overall amino acid profile of BSF larvae in this study exhibited similarity to that of larvae commonly used for animal feed, such as fish, suggesting their potential as a sustainable protein source [16,73].

After subjecting BSF larvae to a diet containing Cd, Pb, and zinc (Zn), Diener et al. [20] investigated the accumulation patterns of these heavy metals. They observed that while Cd accumulated in BSF larvae and pupae, the accumulation of Pb and Zn was suppressed. Although the Pb concentration in larvae and pupae was low, Diener et al. [20] confirmed Pb accumulation in larval exudate. Previous studies have also highlighted the remarkable resistance of BSF larvae to artificial diets containing complex heavy metals [20,24]. Principal component analysis revealed that high concentrations of Pb, nickel, and boron potentially impede larval body weight gain, whereas copper, chromium, Zn, Cd, and Hg appeared to slightly reduce larval survival [24]. In alignment with Diener et al. [20], our study on BSF larvae demonstrated efficient reduction in Pb and Cd, achieving up to a 63% reduction in Pb and a remarkable 430% reduction in Cd when fed with 50% MH. The reduction percentage for Pb and Cd increased with a higher percentage of MH in the BSF larval feed. However, contrasting results were obtained for As and Hg, showing negative outcomes in feeds with higher MH concentrations. Concerning As, the reduction in accumulation through fecal excretion was relatively small compared to other heavy metals. This phenomenon may be attributed to the presence of specific inorganic salts (such as calcium, iron, etc.) and vitamins in BSF larvae, along with the molting and eclosion processes [74]. Consequently, additional experiments are warranted to determine optimal reduction strategies for As and Hg. Further research is essential to establish a comprehensive heavy metal emission model and to comprehend the mechanisms of heavy metal excretion, as well as the effects of nutrients on heavy metal reduction.

Gut loading refers to the practice of providing insects with a specialized diet shortly before they are consumed [75]. This diet is generally enriched with specific nutrients, ensuring that when the insect is eaten, those nutrients are present in its gut. Consequently, the creature consuming the insect receives an increased intake of nutrients [76]. Studies have yielded disparate findings concerning the optimal gut-loading periods and the ideal duration for effectively gut-loading insects, exhibiting slight variations likely attributable to the specific insect species under scrutiny, the palatability of the gut-loading diet, and prevailing environmental conditions such as temperature, light, and humidity [77]. Generally, a gut-loading duration spanning 24 to 72 h appears to generate comparable nutrient levels within the intact insect. Prolonged application of gut-loading diets, however, has been associated with detrimental effects on insect viability [78]. In addition, Anderson [79] has emphasized that the physical composition of the diet and the presence of other nutrients such as amino acids and fatty acids, influencing diet palatability, must be considered when formulating a gut-loading regimen. The incorporation of PUFA into the gut-loading diet has been proposed for insectivores originating from temperate climates, anticipating encounters with insects bearing relatively elevated concentrations of PUFA [80]. In our investigation, we observed that gut-loading BSF larvae with a diet rich in fatty acids resulted in a higher proportion of PUFA, including EPA and DHA, along with other essential amino acids crucial for breeding animals.

In some countries, MH is not considered an unwanted by-product and finds utility in various applications, such as bait or fishmeal [5]. However, in the Republic of Korea, fish heads are typically discarded, contributing to the growing issue of fisheries waste. This study addresses this concern by repurposing wasted MH to cultivate BSF larvae, presenting an additional alternative for utilizing fish by-products. This incorporation of MH substantially enhanced the nutritional content of BSF larvae, surpassing even the nutritional content present in MH alone. Notably, the protein content in BSF larvae exceeded that in MH itself. Consequently, this study demonstrated that supplementing BSF larvae feed with MH increases the protein source for larval feed compared to providing it directly as fishmeal. While previous studies on the utilization of fish by-products for BSF larvae have primarily focused on fatty acid alteration and growth performance, our study contributes additional information, particularly regarding the conversion efficiency and by-product reduction efficiency of BSF larvae. It is essential to note, however, that the applicability of this study may be limited in some countries due to regulations prohibiting the use of former foodstuffs containing meat or fish as feed materials for insect diets [81,82]. The stability of the prepared diet for several generations of insect rearing also remains uncertain, necessitating further detailed studies to assess its viability over extended periods of BSF larvae cultivation.

Human activities are significantly decreasing environmental quality and biodiversity, with interactions historically following a linear economy model of extraction, production, and waste disposal, no longer sustainable for Earth’s capacity [83,84,85,86]. Increased global food demand, particularly for seafood, driven by population growth and dietary trends, intensifies the buildup of fisheries waste. Solutions lie in adopting life cycle thinking and circular economy principles, as advocated by the EU’s Circular Economy Action Plan, to enhance seafood production efficiency and mitigate environmental impact [84]. Valorization strategies and nutrient recovery technologies are crucial in this shift, aiming to identify value in lost materials and promote sustainable business models [87]. Utilizing seafood by-products, such as mackerel heads, can contribute to reducing waste and advancing circular economy goals.

## 5. Conclusions

The current study underscores the potential of MH as a substrate for rearing BSF larvae, with a proportion of 20% in BSF larvae feed proving to be optimal for enhanced growth, efficient waste conversion, and improved nutritional quality. These findings highlight the valuable role of BSF larvae in agriculture and waste management, converting organic waste into nutrient-rich biomass. This study also emphasizes the significance of balanced MH content in the BSF larvae diet, while cautioning against excessively high MH proportion that may hinder growth efficiency. Furthermore, MH supplementation contributing to higher PUFA content further enhances the nutritional value of BSF larvae. Overall, the findings of this study demonstrate the potential of BSF larvae in sustainable waste management and nutrient-rich biomass production.

## Figures and Tables

**Table 1:** Proximate composition of BSF larvae feed containing various proportions of mackerel head.

MH (%)	Moisture (%)	Protein (%)	Lipid (%)	Ash (%)
0	60.22 ± 1.41	8.54 ± 0.02	2.70 ± 0.02	3.10 ± 0.25
10	60.47 ± 0.35	9.82 ± 0.02	4.17 ± 0.04	3.35 ± 0.11
20	60.74 ± 0.43	10.72 ± 0.03	5.75 ± 0.04	3.29 ± 0.01
30	60.46 ± 0.99	11.66 ± 0.03	7.58 ± 0.04	3.96 ± 0.66
40	60.79 ± 0.80	12.57 ± 0.03	9.50 ± 0.26	3.94 ± 0.52
50	60.41 ± 1.02	14.07 ± 0.04	11.37 ± 0.10	4.22 ± 0.11

BSF larvae: Black soldier fly larvae; MH: Mackerel head. Values are expressed as means ± S.E. of 3 individual experiments (*n* = 3).

**Table 2:** Fatty acid content of BSF larvae feed containing various proportions of mackerel head.

Fatty Acid	Proportion of MH in Basal Feed (%)					
	0	10	20	30	40	50
Lauric acid	0.02	0.02	0.01	0.01	0.01	0.01
Myristic acid	0.05	0.12	0.12	0.20	0.21	0.35
Myristoleic acid	0.01	0.01	0.01	0.01	0.01	0.01
Pentadecanoic acid	0.01	0.02	0.02	0.03	0.03	0.05
Palmitic acid	0.76	1.03	1.13	1.42	1.55	2.15
Palmitoleic acid	0.06	0.11	0.13	0.20	0.22	0.36
Margaric acid	0.01	0.03	0.03	0.04	0.04	0.07
Stearic acid	0.30	0.33	0.37	0.40	0.44	0.58
Elaidic acid	0.05	0.06	0.05	0.04	0.05	0.04
Oleic acid	1.10	1.36	1.41	1.55	1.73	2.22
Linoleic acid	0.70	0.68	0.62	0.53	0.47	0.42
Arachidic acid	0.01	0.02	0.02	0.02	0.02	0.03
γ-Linolenic acid	nd	nd	nd	nd	0.01	0.01
cis-11-Eicosenoic acid	0.01	0.05	0.06	0.11	0.11	0.19
α-Linolenic acid	0.02	0.04	0.05	0.06	0.07	0.09
Heneicosanoic acid	nd	nd	0.01	0.01	0.01	0.01
cis-11,14-Eicosadienoic acid	0.01	0.04	0.04	0.09	0.09	0.14
Erucic acid	0.01	0.02	0.02	0.03	0.03	0.04
Arachidonic acid	nd	0.02	0.02	0.04	0.04	0.07
Docosadienoic acid	nd	0.01	0.02	0.03	0.03	0.04
Eicosapentaenoic acid	nd	0.12	0.20	0.29	0.43	0.52
Nervonic acid	nd	0.01	0.01	0.02	0.02	0.04
Docosahexaenoic acid	nd	0.27	0.50	0.75	1.09	1.34

BSF larvae: Black soldier fly larvae; MH: Mackerel head; Fatty acid composition is based on g/100 g lipids extracted from MH; Chicken feed was used as basal feed; nd: not detected.

**Table 3:** Survival rate, feed conversions, and feed reduction of MH-treated BSF larvae.

MH (%)	SR (%)	FCR	ECI (%)	FR (%)
0	99.33 ± 0.67 ^a^	2.50 ± 0.10 ^a^	57.56 ± 2.12 ^a^	69.75 ± 0.46 ^ab^
10	99.67 ± 0.33 ^a^	2.28 ± 0.03 ^b^	61.36 ± 0.85 ^ab^	71.39 ± 0.37 ^ac^
20	99.33 ± 0.67 ^a^	2.09 ± 0.04 ^b^	65.12 ± 1.19 ^bc^	73.53 ± 0.59 ^c^
30	92.67 ± 1.20 ^b^	2.11 ± 0.01 ^b^	64.26 ± 0.80 ^bc^	73.81 ± 1.17 ^c^
40	89.33 ± 1.20 ^c^	2.17 ± 0.04 ^b^	68.88 ± 1.37 ^c^	66.94 ± 1.03 ^bd^
50	81.67 ± 1.20 ^d^	2.50 ± 0.10 ^a^	61.88 ± 3.42 ^ab^	65.08 ± 1.68 ^d^

MH: Mackerel head; BSF larvae: Black soldier fly larvae; SR: Survival rate; FCR: Feed conversion ratio; ECI: Efficiency of ingested feed; FR: Feed reduction. Values are expressed as means ± S.E. of three replicates (*n* = 3). Different superscript letters (a–d) in the same column indicate significant differences among treatments at *p* < 0.05, according to LSD test.

**Table 4:** Proximate composition of MH-treated BSF larvae.

MH (%)	Lipid (%)	Protein (%)	Ash (%)
0	33.65 ± 0.06 ^a^	40.98 ± 0.11 ^a^	6.47 ± 0.09 ^a^
10	35.80 ± 0.11 ^b^	41.37 ± 0.17 ^ab^	6.10 ± 0.06 ^b^
20	37.70 ± 0.10 ^c^	41.81 ± 0.34 ^abc^	5.77 ± 0.03 ^c^
30	37.45 ± 0.63 ^bc^	41.49 ± 0.80 ^abc^	5.70 ± 0.06 ^c^
40	37.74 ± 0.02 ^c^	42.28 ± 0.08 ^bc^	5.30 ± 0.06 ^d^
50	37.45 ± 0.68 ^bc^	42.69 ± 0.13 ^c^	4.90 ± 0.06 ^e^

MH: Mackerel head; BSF larvae: Black soldier fly larvae. Values are expressed as means ± S.E. of three replicates (*n* = 3). Different superscript letters (a–e) in the same column indicate significant differences among treatments at *p* < 0.05, according to LSD test.

**Table 5:** Fatty acid composition of MH-treated BSF larvae (% of lipid extract).

Fatty Acid	Proportion of MH in Basal Feed (%)					
	0	10	20	30	40	50
Capric acid	1.17	0.99	0.91	0.92	0.97	0.89
Lauric acid	30.81	24.42	23.08	22.15	22.19	19.92
Tridecanoic acid	0.00	0.00	0.00	0.00	0.08	0.08
Myristic acid	5.81	5.60	5.92	6.18	6.45	6.53
Pentadecanoic acid	0.12	0.28	0.42	0.55	0.64	0.73
Palmitic acid	15.54	17.81	19.07	19.97	20.76	22.00
Margaric acid	0.28	0.47	0.61	0.72	0.79	0.87
Stearic acid	5.38	5.72	5.51	5.23	4.97	5.04
Arachidic acid	0.13	0.00	0.00	0.00	0.00	0.00
Heneicosanoic acid	0.79	0.28	0.20	0.18	0.30	0.19
Myristoleic acid	0.13	0.12	0.10	0.11	0.09	0.11
Palmitoleic acid	2.31	2.97	3.53	4.08	4.52	4.96
Elaidic acid	0.50	0.43	0.36	0.31	0.28	0.24
Oleic acid	21.80	22.73	21.93	21.56	20.29	20.37
Eicosenoic acid	0.12	0.37	0.58	0.80	0.90	0.97
Linoelaidic acid	0.00	0.00	0.52	0.38	0.47	0.56
Linoleic acid	14.35	13.31	10.88	9.31	7.59	6.80
γ-Linolenic acid	0.00	0.00	0.09	0.11	0.08	0.14
α-Linolenic acid	0.68	0.92	0.99	1.15	1.20	1.35
Arachidonic acid	0.00	0.27	0.37	0.48	0.55	0.59
Eicosapentaenoic acid	0.00	2.26	3.19	3.76	4.30	4.78
Docosahexaenoic acid	0.00	1.05	1.74	2.06	2.57	2.89
ΣSFA	60.09	55.58	55.72	55.90	57.15	56.25
ΣMUFA	24.87	26.62	26.50	26.86	26.09	26.64
ΣPUFA	15.03	17.81	17.79	17.24	16.75	17.11
EPA + DHA	0.00	3.31	4.93	5.81	6.87	7.67

MH: Mackerel head; BSF larvae: Black soldier fly larvae; 0–50% are mackerel head concentrations in basal feed (chicken feed); ΣSFA: Total amount of saturated fatty acids; ΣMUFA: Total amount of monounsaturated fatty acids; ΣPUFA: Total amount of polyunsaturated fatty acids; EPA: Eicosapentaenoic acid; DHA: Docosahexaenoic acid.

**Table 6:** Amino acid composition of MH-treated BSF larvae (g/kg dry matter basis).

Amino Acid	Proportion of MH in Basal Feed (%)		
	0	20	50
Alanine	43.7	40.5	42.6
Arginine	12.0	13.9	11.6
Aspartic acid	28.3	29.8	25.8
Cysteine	4.7	5.1	4.3
Glutamic acid	45.6	47.2	46.0
Glycine	21.8	21.3	21.7
Histidine	10.2	11.2	10.4
Isoleucine	17.1	16.2	16.9
Leucine	27.4	27.0	26.6
Lysine	23.8	25.1	25.8
Methionine	7.8	8.1	7.4
Phenylalanine	16.3	16.5	15.9
Proline	23.0	23.3	23.3
Serine	13.4	14.7	12.4
Threonine	15.9	16.0	15.2
Tryptophan	4.2	4.2	3.5
Tyrosine	17.7	18.4	16.9
Valine	24.4	23.6	23.6

MH: Mackerel head; BSF larvae: Black soldier fly larvae; 0, 20, and 50% are mackerel head concentrations in basal feed (chicken feed).

**Table 7:** Heavy metal content and heavy metal reduction percentage in BSF larvae.

Heavy Metal	MH (%)	Heavy Metal Content (mg/kg)							MR (%)	Standard *
		M_CF_	M_MH_	M_SBSFL_	M_TF_	M_BSFLD_	M_BSFL_	M_BSFL_ − M_SBSFL_		
Pb	0	0.143 ± 0.001	0.046 ± 0.001	0.263 ± 0.009	0.0100	0.099	0.148 ± 0.007 ^a^	−0.114	56.20	0.5
	10				0.0101	0.124	0.117 ± 0.004 ^b^	−0.145	55.97	
	20				0.0098	0.220	0.098 ± 0.005 ^c^	−0.164	43.84	
	30				0.0093	0.109	0.078 ± 0.016 ^d^	−0.184	64.84	
	40				0.0088	0.132	0.060 ± 0.003 ^e^	−0.202	62.20	
	50				0.0082	0.132	0.048 ± 0.008 ^e^	−0.214	63.35	
As	0	0.065 ± 0.011	0.203 ± 0.081	0.253 ± 0.015	0.0046	0.001	0.025 ± 0.007 ^a^	−0.227	97.16	0.5
	10				0.0069	0.028	0.085 ± 0.014 ^a^	−0.167	84.31	
	20				0.0090	0.058	0.128 ± 0.025 ^a^	−0.124	67.77	
	30				0.0111	0.070	0.210 ± 0.059 ^a^	−0.042	38.01	
	40				0.0132	0.111	0.275 ± 0.086 ^a^	0.023	−25.68	
	50				0.0150	0.182	0.352 ± 0.107 ^a^	0.100	−129.77	
Hg	0	0.010 ± 0.001	0.030 ± 0.001	0.054 ± 0.008	0.0007	0.001	0.004 ± 0.002 ^d^	−0.053	20.39	0.05
	10				0.0010	0.003	0.016 ± 0.001 ^cd^	−0.041	16.49	
	20				0.0014	0.004	0.027 ± 0.007 ^bc^	−0.030	12.60	
	30				0.0017	0.005	0.037 ± 0.001 ^ab^	−0.020	8.54	
	40				0.0020	0.007	0.042 ± 0.004 ^ab^	−0.015	6.44	
	50				0.0022	0.010	0.049 ± 0.007 ^a^	−0.008	3.39	
Cd	0	0.005 ± 0.001	0.041 ± 0.001	0.758 ± 0.062	0.0004	0.036	0.137 ± 0.019 ^a^	−0.652	403.08	0.5
	10				0.0008	0.039	0.117 ± 0.018 ^a^	−0.671	365.59	
	20				0.0013	0.015	0.109 ± 0.010 ^a^	−0.679	407.30	
	30				0.0018	0.020	0.107 ± 0.017 ^a^	−0.681	393.53	
	40				0.0023	0.022	0.112 ± 0.032 ^a^	−0.676	398.32	
	50				0.0027	0.026	0.134 ± 0.059 ^a^	−0.654	430.28	

Values are expressed as means ± S.E. of three replicates (*n* = 3). Pb: Lead; As: Arsenic; Hg: Mercury; Cd: Cadmium; MH: Mackerel head; BSF larvae: Black soldier fly larvae; M_CF_: Heavy metals in chicken feed; M_MH_: Heavy metals in Mackerel head; M_SBSFL_: Heavy metals in BSF larvae before starting the feeding experiments; M_TF_: Heavy metals in chicken feed supplemented with 0 to 50% (*w*/*w*) MH; M_BSFLD_: Heavy metals in the feces of BSF larvae; M_BSFL_: Heavy metals in BSF larvae that consumed chicken feed containing 0 to 50% (*w*/*w*) MH, fasted, and with feces removed; MR %: Heavy metal reduction percentage. Different superscript letters (a–e) in the same column indicate significant differences among treatments at *p* < 0.05, according to LSD test. * The acceptable standards for hazardous substances in ‘fisheries products’ mentioned in the April 2017 re-evaluation report on heavy metal standards for food [36].

## Data Availability

The data presented in this study are available on request from the corresponding author. The data are not publicly available due to a patent application linked with the study outcomes.
